# Epidemiology of Trauma-Related Hemorrhage and Time to Definitive Care Across North America: Making the Case for Bleeding Control Education

**DOI:** 10.1017/S1049023X23006428

**Published:** 2023-12

**Authors:** Allison R. Jones, Justin Miller, Michelle Brown

**Affiliations:** 1. Department of Acute, Chronic & Continuing Care, School of Nursing, University of Alabama at Birmingham, Birmingham, Alabama USA; 2. Department of Health Services Administration, School of Health Professions, University of Alabama at Birmingham, Birmingham, Alabama USA

**Keywords:** emergency treatments, hemorrhage, injury, trauma, wound

## Abstract

**Introduction:**

Uncontrolled trauma-related hemorrhage remains the primary preventable cause of death among those with critical injury.

**Study Objective:**

The purpose of this investigation was to evaluate the types of trauma associated with critical injury and trauma-related hemorrhage, and to determine the time to definitive care among patients treated at major trauma centers who were predicted to require massive transfusion.

**Methods:**

A secondary analysis was performed of the Pragmatic, Randomized, Optimal Platelet and Plasma Ratios (PROPPR) trial data (N = 680). All patients included were predicted to require massive transfusion and admitted to one of 12 North American trauma centers. Descriptive statistics were used to characterize patients, including demographics, type and mechanism of injury, source of bleeding, and receipt of prehospital interventions. Patient time to definitive care was determined using the time from activation of emergency services to responder arrival on scene, and time from scene departure to emergency department (ED) arrival. Each interval was calculated and then summed for a total time to definitive care.

**Results:**

Patients were primarily white (63.8%), male (80.3%), with a median age of 34 (IQR 24-51) years. Roughly one-half of patients experienced blunt (49.0%) versus penetrating (48.2%) injury. The most common types of blunt trauma were motor vehicle injuries (83.5%), followed by falls (9.3%), other (3.6%), assaults (1.8%), and incidents due to machinery (1.8%). The most common types of penetrating injuries were gunshot wounds (72.3%), stabbings (24.1%), other (2.1%), and impalements (1.5%). One-third of patients (34.5%) required some prehospital intervention, including intubation (77.4%), chest or needle decompression (18.8%), tourniquet (18.4%), and cardiopulmonary resuscitation (CPR; 5.6%). Sources of bleeding included the abdomen (44.3%), chest (20.4%), limb/extremity (18.2%), pelvis (11.4%), and other (5.7%). Patients waited for a median of six (IQR4-10) minutes for emergency responders to arrive at the scene of injury and traveled a median of 27 (IQR 19-42) minutes to an ED. Time to definitive care was a median of 57 (IQR 44-77) minutes, with a range of 12-232 minutes. Twenty-four-hour mortality was 15% (n = 100) with 81 patients dying due to exsanguination or hemorrhage.

**Conclusion:**

Patients who experience critical injury may experience lengthy times to receipt of definitive care and may benefit from bystander action for hemorrhage control to improve patient outcomes.

## Introduction

Trauma claims the lives of more than four million people world-wide annually with uncontrolled hemorrhage being a primary cause of death among those injured.^1,2^ In the United States alone, trauma remains a leading cause of death for people of all ages, with the greatest impact on adults under the age of 45 and adolescents.^3^ Importantly, almost one-quarter of deaths due to uncontrolled hemorrhage may have been prevented.^4^ Control of active bleeding requires immediate intervention commonly provided through the application of pressure, packing open wounds, and/or application of tourniquets.

**Figure 1.**
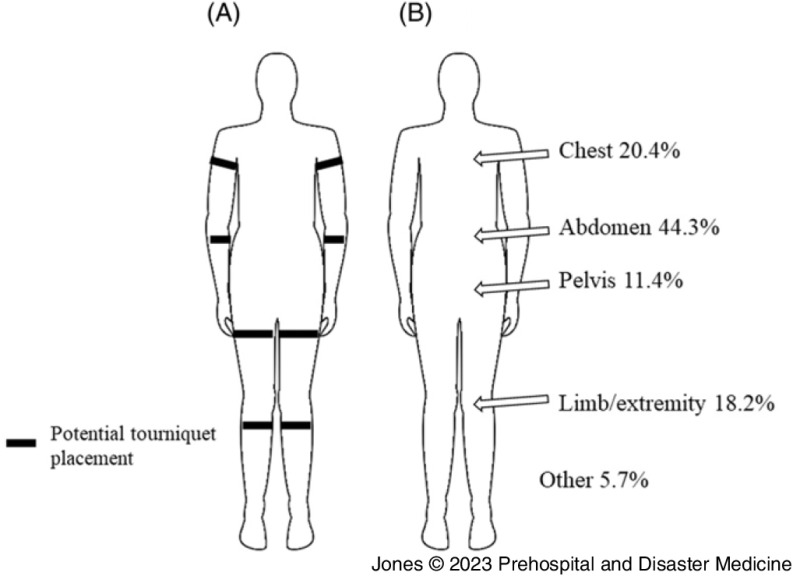
**(A)** Depiction of Anatomical Locations where Tourniquets may be Applied. Wounds in Other Areas must be Treated with Manual Pressure and/or Wound Packing Only. **(B)** Sources of Bleeding among Patients.

Outcomes for patients with trauma depend largely on the anatomic location of wounds, extent of injury, the prompt arrival of emergency responders, and efficient transport to definitive care. In situations where injuries are witnessed, opportunity exists for bystanders to intervene. The Stop the Bleed (STB) campaign is a public health initiative through the United States Department of Homeland Security (DHS; Washington, DC USA) that started in 2015 under the Obama administration, the purpose of which is to promote early bystander intervention and reduce mortality due to injury and uncontrolled hemorrhage.^5^ The STB training includes instruction on how to identify life-threatening bleeding, locating the source of the bleeding, and methods for bleeding control based on the type and location of the wound (eg, tourniquets may only be used on extremities; Figure 1A). While the STB initiative was born out of concern for the increase in mass-casualty events such as shootings, the essentials of bleeding control may be applied to any scenario involving hemorrhage due to trauma.

Evidence continues to emerge supporting the influence of bystander intervention in improving survival outcomes in patients who experience critical health events such as cardiac arrest.^6–8^ In contrast to the American Heart Association’s (AHA; Dallas, Texas USA) Basic Life Support training established in the 1980s,^9^ STB remains in its infancy and is largely unknown to the lay public. Determination of the most common types of trauma and those that require immediate attention for bleeding control could help to inform and recruit trainees and prepare them to act and may lead to further development of interventions to promote bleeding control education among the lay public. Thus, the purpose of this investigation was to perform an epidemiological evaluation of the types of trauma most associated with the need for hemorrhage control and large volume transfusion, and to determine the time to definitive care for those with critical injury.

## Materials and Methods

### Study Design and Setting

A secondary analysis was performed of de-identified data from the Pragmatic, Randomized, Optimal Platelet and Plasma Ratios (PROPPR) trial.^10^ This protocol was reviewed and approved by the University of Alabama at Birmingham (Birmingham, Alabama USA) Institutional Review Board (#300006932).

### Selection of Participants

All patients (N = 680) were included who were recruited from one of 12 North American Level I trauma centers and met the following inclusion criteria: experienced severe injury requiring the highest level of trauma activation based on local criteria; received at least one unit of any blood component prior to hospital arrival or within one hour of admission; were predicted to require massive transfusion based on an Assessment of Blood Consumption score of two or greater, or by physician judgment of the need for massive transfusion (10 or more units of packed red blood cells in a 24-hour period); estimated age of 15 or older or weight of 50kg or greater if age unknown; and received directly from the scene of injury.

### Measurements

Patient demographics of age, sex, and race were collected. Clinical variables included mechanism of injury (blunt versus penetrating, or both), prehospital interventions (intubation, tourniquet, cardiopulmonary resuscitation [CPR], and chest/needle decompression), and source of bleeding (abdomen, chest, intracranial, limb/extremity, neck, pelvis, and scalp/face). Time to definitive care was broken down into two intervals: time from Emergency Medical Services (EMS) activation or call to EMS arrival on scene, and time from EMS arrival on scene to patient arrival in the emergency department (ED). Times from the two intervals were summed to create a total time to definitive care. Mortality status was recorded at 24-hours post-hospital admission.

### Statistical Analysis

Descriptive statistics of median and interquartile range (IQR), and frequency and percentage, were used to characterize the sample and present time intervals. Analyses were completed using Stata release 17 (StataCorp LLC; College Station, Texas USA).^11^

## Results

Patients were primarily white (63.8%), male (80.3%), with a mean age of 38 years (standard deviation [SD] = 17); Table 1. Roughly one-half of patients experienced blunt (49.0%) versus penetrating (48.2%) injury. The most common types of blunt trauma were motor vehicle injuries (83.5%), followed by falls (9.3%), with assaults, incidents due to machinery, and “other” comprising the remainder. The most common types of penetrating injuries were gunshot wounds (72.3%) and stabbings (24.1%). More than one-third of patients (34.5%) required at least one prehospital intervention, including intubation (77.4%), chest or needle decompression (18.8%), tourniquet placement (18.4%), or CPR (5.6%). Sources of bleeding included the abdomen (44.3%), chest (20.4%), limb/extremity (18.2%), pelvis (11.4%), and other (5.7%); Figure 1B. Patients waited for a median of six (interquartile range [IQR] 4-10, range 0-72) minutes for emergency responders to arrive at the scene of injury and traveled a median of 27 (IQR 19-42, range 1-168) minutes to an ED (Table 2). Time to definitive care was a median of 57 (IQR 44-77) minutes with a range of 12-232 minutes. Twenty-four-hour mortality was 15% (n = 100). Of those who died, 81% (n = 81) of patients died due to exsanguination or hemorrhage.

**Table 1.** Patient Characteristics (N = 680)

Characteristic	Value
Age (years)	38 (SD = 17)
Race	
White	434 (63.8)
Black	186 (27.4)
Other	60 (8.8)
Male	546 (80.3)
Mechanism of Injury^a^	
Blunt	333 (49.0)
MVC (Motorcycle, Bicycle, Occupant, Pedestrian, Other, Unknown)	278 (83.5)
Assault	6 (1.8)
Fall	31 (9.3)
Machinery	6 (1.8)
Other	12 (3.6)
Penetrating	328 (48.2)
Gunshot Wound	237 (72.3)
Impalement	5 (1.5)
Stabbing	79 (24.1)
Other	7 (2.1)
Blunt & Penetrating	8 (1.2)
Prehospital Interventions^a^	234 (34.5)
Intubation	181 (77.4)
Tourniquet	43 (18.4)
Cardiopulmonary Resuscitation	13 (5.6)
Chest/Needle Decompression	44 (18.8)
Source of Bleeding^b^ (n = 456)	
Abdomen	202 (44.3)
Chest	93 (20.4)
Intracranial	8 (1.8)
Limb/Extremity	83 (18.2)
Neck	10 (2.2)
Pelvis	52 (11.4)
Scalp/Face	8 (1.8)
24-Hour Mortality	100 (14.7)
Cause - Exsanguination/Hemorrhage	81 (81.0)

Abbreviation: MVC, motor vehicle collision.

a More than one category may apply.

b Numbers may not equal 100% due to missing data.

**Table 2.** Time to Definitive Care

Interval^a^	Value
Time from EMS Call to Arrival On Scene (n = 545)	6 (4-10) minutes, range 0-72
Time from EMS Arrival On Scene to ED Arrival (n = 551)	27 (19-42) minutes, range 1-168
Total Time to Definitive Care (n = 545)	57 (44-77) minutes, range 12-232

Note: Definitive care is defined as care established in the emergency department.

Abbreviations: EMS, Emergency Medical Services; ED, emergency department.

a All times reported in minutes using median (interquartile range) and range (minimum to maximum).

## Discussion

In the present study, it was found that the most common cause of blunt trauma included motor vehicle crashes and of penetrating trauma included gunshot wounds/stabbings. The source of hemorrhage for most patients was the abdomen, chest, and/or a limb/extremity. Roughly one-third of patients required at least one prehospital intervention including tourniquet placement for hemorrhage control. Patients in the current study also experienced a median wait time of six minutes for EMS to arrive and a median of 60 minutes until reaching definitive care, presenting a significant delay in care.

These findings are consistent with the existing, limited epidemiological studies on trauma and support the need for early bystander intervention. The National Academies of Sciences (NASEM; Washington, DC USA) estimated that 20% of trauma-related deaths may have been preventable with receipt of “optimal trauma care” and noted that the “greatest opportunity to save lives” is in the prehospital setting.^12^ In a recent analysis of 1,848 trauma-related deaths, Kalkwarf, et al found that 305 were due to uncontrolled hemorrhage.^13^ They further reported that 45% of deaths (n = 137) were preventable or potentially preventable, with 35% of these deaths occurring in the prehospital setting.^13^ In a separate investigation of the same data set, other investigators reported that among 847 prehospital deaths, 89 may have been prevented, and of these, 55% were due to hemorrhage.^14^

Patients with life-threatening hemorrhage may lose their entire circulating blood volume in under five minutes,^15^ making immediate intervention crucial to their survival. While injuries to the abdomen and chest generally require surgical intervention to control bleeding, limb/extremity wounds and those to other areas of the body may benefit from bystander intervention such as that taught in the STB courses.^2,16^ Despite its simplicity and wide-spread applicability, STB training is still in its infancy and remains largely unknown to the lay public. To put this in perspective, the American Heart Association alone trains more than 22 million people globally in CPR *each year*.^9^ Findings from an analysis of 110,054 witnessed out-of-hospital cardiac arrests highlight the substantial effect of bystander intervention, as 47% of those who provided CPR were laypersons.^17^ Further, investigators of a recent systematic review including 19 studies and more than 230,000 patients found that patients with witnessed out-of-hospital cardiac arrests who received bystander CPR were nearly two-times more likely to survive compared to those who did not receive bystander CPR (pooled odds ratio 1.95, 95% confidence interval [CI]: 1.66–2.30).^18^ In contrast, less than 2.5 million people world-wide have *ever* received STB training.^19^ This disparity in emergency response trainings presents a significant opportunity to reduce mortality and improve outcomes following major trauma and unintentional injury.

Though refresher training is required every two years to maintain CPR certification,^20^ no recommendations or requirements for STB skill maintenance currently exist. Recent evidence suggests that average rates of correct tourniquet application fall from 100% after initial training to 69% only six months after training (P <.001); however, only 46 participants were included in the study.^21^ Similarly, Goralnick, et al conducted a randomized clinical trial among laypersons (n = 465) to test the effectiveness of instructional point-of-care interventions and in-person training for hemorrhage control training compared to no intervention.^22^ At three-to-nine months after training, investigators assessed the retention of skills for 303 participants and found that 54.5% could correctly apply a tourniquet.^22^ Though striking, these findings represent small samples and additional research is required to further examine retention rates and factors associated with increased skill retention.

Feedback from STB participants across multiple studies indicate an appreciation of the significance of bleeding control training, confidence in the ability to intervene when necessary and willingness to do so, and the desire for regular refresher training.^23–26^ Despite good intentions and willingness to help those injured, barriers to the provision of bleeding control aid in the prehospital setting exist among the lay community and may include: lack of access to supplies such as tourniquets and hemostatic gauze to effectively stop bleeding, fear of disease transmission on the part of the bystander without the use of gloves, fear of inflicting additional pain, fear of being sued by the injured person, concern for lack of physical ability to completely stop active bleeding, and fear of wound contamination or additional tissue damage with tourniquet application.^27^ Importantly, the extent to which these or any additional barriers may exist, how they may most effectively be addressed, and the impact they have on bystander intervention have yet to be fully elucidated.

## Limitations

Findings from the present study must be considered in light of several limitations. While the data in this secondary analysis represent adult patients with major trauma treated at 12 Level I trauma centers across North America, details on locations of traumas and EMS (distance to the scene, personnel staffing, and experience of the providers) in the geographic location were not included. Therefore, the analyses were unable to be stratified based on urban versus rural locations. Circumstances surrounding the trauma may also have influenced the time to definitive care but were not available for analysis. For example, patients in motor vehicle accidents who required extrication may have experienced prolonged delays in treatment and hospital arrival. Finally, these data were collected as part of a clinical trial to examine the impact of ratios of blood components used in massive transfusions for patients with major trauma and were therefore not powered to address researcher questions.

## Conclusions

Traumatic injuries occur frequently and patients with severe hemorrhage require immediate intervention to control active bleeding. The epidemiological evaluation of the types of trauma most associated with the need for hemorrhage control and the time to definitive care among patients seen in major trauma centers across North America supports the need for STB training among bystanders. The promotion of bleeding control training among lay community members may increase bystander intervention and reduce mortality. Further research is needed to explore facilitators and barriers to implementing bleeding control education in community settings, factors associated with skill retention, and methods to promote bleeding control aid among bystanders.
